# Prioritizing the scale-up of interventions for malaria control and elimination

**DOI:** 10.1186/s12936-019-2755-5

**Published:** 2019-04-08

**Authors:** Peter Winskill, Patrick G. Walker, Richard E. Cibulskis, Azra C. Ghani

**Affiliations:** 1 MRC Centre of Global Infectious Disease Analysis, School of Public Health, Faculty of Medicine, Imperial College London, St Mary’s Campus, London, W2 1PG UK; 2 Global Malaria Programme, World Health Organization, Geneva, Switzerland

**Keywords:** Malaria, *Plasmodium falciparum*, Interventions, Prioritization, Cost-effective, Scale-up

## Abstract

**Background:**

A core set of intervention and treatment options are recommended by the World Health Organization for use against falciparum malaria. These are treatment, long-lasting insecticide-treated bed nets, indoor residual spraying, and chemoprevention options. Both domestic and foreign aid funding for these tools is limited. When faced with budget restrictions, the introduction and scale-up of intervention and treatment options must be prioritized.

**Methods:**

Estimates of the cost and impact of different interventions were combined with a mathematical model of malaria transmission to estimate the most cost-effective prioritization of interventions. The incremental cost effectiveness ratio was used to select between scaling coverage of current interventions or the introduction of an additional intervention tool.

**Results:**

Prevention, in the form of vector control, is highly cost effective and scale-up is prioritized in all scenarios. Prevention reduces malaria burden and therefore allows treatment to be implemented in a more cost-effective manner by reducing the strain on the health system. The chemoprevention measures (seasonal malaria chemoprevention and intermittent preventive treatment in infants) are additional tools that, provided sufficient funding, are implemented alongside treatment scale-up. Future tools, such as RTS,S vaccine, have impact in areas of higher transmission but were introduced later than core interventions.

**Conclusions:**

In a programme that is budget restricted, it is essential that investment in available tools be effectively prioritized to maximize impact for a given investment. The cornerstones of malaria control: vector control and treatment, remain vital, but questions of when to scale and when to introduce other interventions must be rigorously assessed. This quantitative analysis considers the scale-up or core interventions to inform decision making in this area.

**Electronic supplementary material:**

The online version of this article (10.1186/s12936-019-2755-5) contains supplementary material, which is available to authorized users.

## Background

The World Health Organization (WHO) Global Technical Strategy for Malaria 2016–2030 (WHO-GTS) sets the goal of universal access to malaria prevention, treatment and diagnosis [1]. This includes a core package of recommended interventions for reducing malaria-related morbidity and mortality: diagnosis and treatment of clinical and severe malaria, vector control with long-lasting insecticide-treated bed nets (LLINs) or indoor residual spraying (IRS) and chemoprevention for high-risk groups (infants, children in areas of seasonal transmission, pregnant women). The costs of providing these core interventions to all individuals residing in areas of malaria risk were estimated at US$2.7 billion per year in 2016, which will rise to $6.4 billion (95% UI US$4.5–9.0 billion) by 2020 if intermediate scale-up targets on the road to universal coverage are reached [1–3]. This remains significantly higher than the current estimated malaria spend of US$2.5–3 billion per year since 2010 [3]. Given this funding gap, both country-level and international decisions regarding which interventions to fund and where are shaped by the shortfalls in required funds.

Ensuring universal access to prompt and effective diagnosis and treatment of malaria is both a health system priority as well as an ethical goal [1]. In the majority of malaria-endemic countries, artemisinin-based combination therapy (ACT) is used for effective first-line treatment of uncomplicated cases of falciparum malaria and intravenous artesunate for severe malaria. Vector control is implemented in all malaria-endemic countries, with the specific intervention mix between LLINs, IRS and other methods influenced by the distribution, relative abundance and behaviour of different vector species, as well as other context-specific factors such as the historical use of a particular form of vector control. The WHO recommends universal coverage of the population at risk with either LLINs or IRS [1] and whilst a combination of the two is deployed in some areas (primarily for insecticide resistance management), WHO recommends attaining high coverage of a single method before a second form is deployed.

In areas of moderate to high transmission, chemoprevention of high-risk groups is recommended. This includes intermittent preventive treatment of infants (IPTi), intermittent preventive treatment of pregnant women (IPTp) and seasonal malaria chemoprevention (SMC) for children under 5 years in highly seasonal areas of the Sahel region [1]. Although not currently recommended by WHO, the RTS,S vaccine is a potential additional tool that is in the process of large-scale post phase III pilot implementation in Ghana, Kenya and Malawi [4, 5].

Whilst coverage and access for these core recommended interventions have increased over time, significant gaps still exist. In 2015, it was estimated that only 19.7% (95% CI 15.6–24.8%) of febrile children under 5 years with *Plasmodium falciparum* received ACT [6]. In 2016 in sub-Saharan Africa 54% (95% CI 50–58%) of the population at risk slept under an insecticide-treated bed net (ITN) [3]. The proportion of the population at risk protected by IRS has declined from 5.8% in 2010 to 2.9% in 2016 [3]. Of the recommended chemoprevention measures, SMC has been implemented at scale and at high coverage in a sub-set of eligible areas [3, 7], IPTi has yet to be implemented programmatically at scale [3] and the coverage of three or more doses of IPTp remains less than 20% [3].

These interventions constitute a core cost-effective toolkit for malaria control [8]. LLINs remain a highly cost-effective intervention and their mass production and distribution has led to falling costs since their introduction [8, 9]. Cost-effective prevention is coupled with cost-effective front-line treatment in the form of ACT [8]. The chemo-preventative interventions have been shown to be highly cost-effective in a number of settings, a trend largely driven by the low cost of drugs recommended for SMC [10], iPTi [11] and IPTp [12]. The RTS,S vaccine has also been estimated to be an additional cost-effective tool (base on an assumed cost-per-dose of $5) in the near future [13].

Despite the availability of cost-effective tools, ambitious coverage targets combined with limited budgets mean that national malaria programmes and other key stakeholders face difficult decisions on where to focus available funds. Being able to effectively prioritize which interventions to fund and on what scale becomes increasingly important in these circumstances. It is therefore helpful for such decisions to be quantitatively assessed to ensure that the maximum marginal impact is gained for the least marginal cost. The best prioritization of interventions that will ensure effective allocation of resources for malaria control and elimination has been considered. By combining data on the efficacy and cost of delivering individual interventions at varying levels of coverage within a mathematical model, estimates were made of the most cost-effective packages of interventions for a given budget in order to maximize the reduction in malaria transmission, case incidence and mortality. The study also highlights the complex interactions between vector control, treatment and the measure of burden.

## Methods

### Transmission model

An individual-based mathematical model of falciparum malaria was used to estimate the impact of different intervention packages across a range of transmission settings [14, 15]. Full mathematical details of the model and its parameterization are given in previous publications [16] and a brief description provided here.

The model considers individuals in a population with the following dynamics. After a period of partial protection due to passively acquired maternal immunity, new-born individuals are susceptible to malaria infection following a bite from an infectious mosquito. After exposure, an infected individual may develop clinical disease which can lead to severe disease and death [17]. Clinical disease may also lead to the individual seeking and receiving treatment that clears the infection (with a given probability), returning the individual to the susceptible state and providing a period of drug-dependent prophylaxis. Those not receiving treatment or who, upon infection, do not present with clinical symptoms, harbour asymptomatic and sub-patent infections before the infection is cleared naturally. The dynamics are strongly influenced by the acquisition of immunity, which is driven by age and/or exposure and impacts the likelihood of infection, detection and/or disease.

The human model is linked to a mosquito population model to allow the impact of vector control to be explicitly captured [18]. Mosquitoes are modelled in four life-stages: eggs and early larval instars, late larval instars, pupae, and adults. Larvae are subjected to density-dependent mortality which governs stability in mosquito population numbers. The model includes a rainfall-dependent carrying capacity allowing dynamics associated with specific seasonal profiles in rainfall to be captured. Species-specific dynamics and bionomics are represented through several parameters quantifying a given species’ propensity to feed on humans, bite indoors and bite at night as well as the relationship between rainfall and density.

The human and mosquito models are linked via the infectivity profiles. The force of infection experienced by humans at each time is determined by the Entomological Inoculation Rate (EIR)—the average number of infectious bites per human per day—which in turn is determined by the prevalence of infection in the adult mosquito population, the human biting rate and the probability that a mosquito survives the extrinsic incubation period. Transmission from humans to mosquitoes is determined by the human infectious reservoir—calculated as a weighted combination of the different infection states (those with clinical disease being the most infectious, patent asymptomatic infection of intermediate infectiousness, and sub-patent infection the lowest infectiousness) and age-based probability of biting (adults receiving more bites than children), and the human biting rate.

Three generic transmissions settings were defined with baseline (in the absence of any interventions or treatment) *Plasmodium falciparum* prevalence in 2 to 10 years old (*Pf*Pr_2-10_) of 10% (low), 30% (medium) and 60% (high) and two generic climatic settings as non-seasonal (perennial transmission, modelled based on rainfall patterns in Equateur Province, DRC) and seasonal (a single seasonal peak, modelled based on rainfall patterns in Upper East Ghana). All sites shared the same distribution of vector species: 50% *Anopheles gambiae s.s.*, 25% *Anopheles funestus* and 25% *Anopheles arabiensis* representing a mixture of the three dominant species observed in sub-Saharan Africa [19].

### Interventions

Intervention packages modelled included combinations of treatment (of clinical and severe malaria), LLINs, SMC, IPTi, and the RTS,S vaccine. Treatment coverage was considered to be the proportion of symptomatic cases that receive the appropriate and effective front-line treatment (ACT or intravenous artesunate). Treatment coverage bands of 25, 50, 65, 75, 85, and 95% were considered. It was assumed that treatment coverage consisted of a mixture of public and private sector where private sector treatments make up 22% of the total on average [the median public sector contribution for countries in sub-Saharan Africa (SSA) (Additional file 1: public private sector treatment)]. The public private sector split remained constant as overall treatment coverage increased (i.e., increases in coverage are absorbed by both the public section (the majority) and the private sector). The recipients of different interventions were assumed to be randomly correlated. The impact of prophylaxis as a result of treatments given to parasite negative cases was not modelled.

LLINs were assumed to be distributed on a 3-year cycle. The modelled impact of LLINs is via both personal protection (a physical barrier to mosquitoes biting individuals sleeping under a net) and community protection (due to the killing effect of the insecticide). Nets were assumed to be effective and not impacted by insecticide resistance. LLIN access bands of 25, 50, 65, 75, 85, and 95% were considered. Due to the low and declining coverage of IRS in SSA [3], it was not included in this analysis.

SMC was modelled as three rounds of sulfadoxine-pyrimethamine + amodiaquine administered to children aged 6 months to 5 years old. The doses were timed with respect to the peak in transmission: the first 1 month prior, the second with the peak and last 1 month post. SMC was evaluated at three high coverage bands (75, 85, 95%), based on levels observed in the ACCESS-SMC implementation programme [7]. SMC was considered in seasonal medium- and high-transmission settings as recommended by the WHO. IPTi was modelled as per WHO recommendations as a full course (3 rounds) of sulfadoxine-pyrimethamine (SP) given to infants during their first year of life in line with the Expanded Programme of Immunization (EPI) schedule. Due to a lack of large-scale programmatic implementation to inform coverage, IPTi was evaluated at the same coverage bands (75, 85, 95%) as SMC. IPTi was considered in non-seasonal medium- and high-transmission settings as recommended by WHO. IPTp was not included in this modelling framework due to the unique dynamics associated with malaria in pregnancy and the challenges of assessing burden and impact across mothers and infants.

### RTS,S

RTS,S was considered at three high coverage bands (75, 85, 95%) in medium- and high-transmission settings. High coverage reflects those consistently observed for routine immunization through the EPI [20]. A four-dose schedule was assumed (at 6, 7.5, 9, and 27 months).

When modelling packages of interventions, the model was run until a new equilibrium level of transmission was reached. The equilibrium was then used to quantify the different impact of intervention packages. This approach provides a standardized method which allows a like-for-like comparison of the impact of different intervention packages. It does however factor out dynamical shifts in transmission that occur before the new steady state is reached due to changes in the interventions used, their coverage and the timings at which changes occur.

### Costs

All costs were estimated from the provider perspective and are summarized in Table 1. Costs are based on two components:

**Table 1:** Intervention and treatment cost components

Cost	Cost (95% CrI)	References
Treatment		
Cost per clinical case under five	$5.36 ($2.34, $13.51)	–
Cost per clinical case over five	$6.26 ($3.24, $14.41)	–
Cost per severe case under five	$31.03 ($5.40, $233.78)	–
Cost per severe case over five	$33.93 ($8.30, $236.86)	–
Treatment component costs		
RDT	$0.60	[2]
ACT under five	$0.30	[23]
ACT over five	$1.20	[23]
Injectable artesunate under five	$1.30	[23]
Injectable artesunate over five	$4.20	[23]
Outpatient visit	$2.61 ($0.61, $11.68)	[24]
Inpatient day stay	$9.91 ($1.37, $77.50)	[24]
Severe malaria hospital stay length	3 days	[2]
NMF per malaria fever	1.86 (0.47, 7.12)	[25]
Health system fixed cost (per capita)	0.20	[26]
Treatment distribution per case (at 50% coverage)	$0.64 ($0.48, $3.01)	[2]
LLINs		
LLIN cost per net	$2.24	[27]
LLIN in-country delivery cost per net delivered	$2.65 ($0.71, $4.61)	[9]
LLIN programme fixed cost per capita per year	$0.20	[26]
SMC		
SMC cost per dose SP-AQ	$0.34	[7]
SMC fixed cost per child per year	$2.13 ($1.00, $4.53)	[7]
SMC variable cost per protected child per year	$2.31 ($1.67, $3.19)	[7]
IPTi		
IPTi cost per dose SP	$0.16	[28] S3
IPTi fixed costs per child per year	$2.13 ($1.00, $4.53)	[7, 28]
IPTi variable costs per protected child per year	$1.15 ($0.93, $1.42)	[7, 28]
RTS,S		
RTS,S cost per dose	$5 (assumed)	[29]
RTS,S fixed cost per child per year	$9.09 ($0.99, $84.20)	[20]
RTS,S variable cost per fully vaccinated child	$33.31 ($5.66, $197.41)	[20]

Where cost uncertainty was propagated, costs are shown as median and 95% credible intervals. Costs for treatment of severe and clinical cases were correlated. RTS,S fixed and variable costs were correlated. Treatment distribution and the number of nets distributed increase non-linearly with respect to coverage

- Fixed costs: These represent programme costs (e.g., recurrent/management/training costs) and do not change with respect to coverage.
- Variable costs: These represent commodity costs (e.g., vaccine dose costs) and increase linearly with coverage.

For LLINs and treatment there is evidence to suggest that there are increasing marginal costs associated with reaching increasingly high coverage. This non-linearity occurs for a number of reasons which include inefficiencies compounding at higher coverage (e.g., over-allocation of bed nets [21]) and that the last mile is the most costly to access (e.g., migrant or rural populations being most isolated from the formal health-system infrastructure [22]). For these interventions a third cost component was included:

c. Variable non-linear costs: These represent inefficiencies or increasing marginal costs as coverage increases. The functional form of non-linear costs was characterized by a fitted hill function (Additional file 1: non-linear fits).

### Treatment

Treatment costs for outpatient visits (clinical episodes) and inpatient hospital stays (severe disease) were estimated using the WHO-CHOICE framework for all counties in SSA [16] (Table 1). Costs include testing using a rapid diagnostic test (RDT) and are adjusted to account for the cost of negative RDT tests due to non-malarial fever. Uncertainty estimates were drawn from a multivariate lognormal fitted to these data to represent the strong correlation between outpatient and inpatient costs. Drug costs scale with coverage and the number of cases. Costs associated with the distribution of and access to treatment were assumed to increase non-linearly with coverage to capture the pattern of increasing marginal cost of achieving high treatment coverage. The uncertainty in the strength of this non-linear relationship increased with respect to coverage after coverage greater than 46% (the maximum currently observed treatment coverage estimated in SSA [6]). Due to a lack of data, a high degree of uncertainty was incorporated in the functional form, ranging from a continuation of linear increases to rapidly increasing (non-linear) costs at higher than 46% coverage (Additional file 9: Fig. S8). A fixed, programmatic cost was also included [26].

### LLINs

LLIN costs were based on estimates of the relationship between the number of bed nets in a population and usage (or access) [21]. This relationship is highly non-linear, accounting for inefficiencies in the system including wastage and over-allocation. The commodity cost of a net was estimated as an average of purchases recorded in the Global Fund Price Reference Report [27] and distribution costs (including uncertainty) from [9] (Table 1).

### SMC

A cost per dose of SP-AQ of $0.34 was used [7] (Table 1). SMC costs consisted of a variable component which scales linearly with coverage and a fixed component which is constant with respect to coverage. Variable costs consisted of reported commodity and distribution costs and fixed costs of reported management, supervision, meetings, training, and recurrent.

### IPTi

Distribution costs were also assumed to be the same as those for SMC. Whilst distribution costs may be shared with the EPI, implementing IPTi would present a departure from standard EPI methodology. As such, a conservative assumption was made that distribution costs would represent a stand-alone programme, such as ACCESS-SMC. Drug costs were rescaled to a value of $0.16 per dose of SP [28] (Table 1).

### RTS,S

The RTS,S vaccine was costed at an estimated $5 per dose [29] (Table 1). Vaccine costs were split into two components, a variable component which scales linearly with coverage and a fixed component which is constant with respect to coverage. Variable costs consisted of vaccine, distribution and commodity costs and fixed costs of reported programme costs. The distribution of costs was estimated from country-level EPI reports [20], scaled to the estimate of RTS,S cost per dose. As with IPTi, and in light of the proposed dosing schedule for the RTS,S vaccine, the conservative assumption was made that there would be minimal shared costs with the EPI programme.

### Estimating the most cost-effective scale-up of interventions

The budget for malaria control was taken to represent funds available for the direct implementation of core interventions, including case management, from both domestic resources and international donors.

The primary public health outcome considered when optimizing scale-up was the mortality rate across all age groups. Secondary analyses with case incidence or an equally weighted combination of case incidence and mortality rate as individual outcomes are included in Additional file 1. These packages differ since prevention interventions impact both case incidence and mortality, whilst treatment primarily influences mortality. Thus, the most cost-effective combination of interventions depends on the outcome measure considered.

The most cost-effective scale-up of interventions was determined by the incremental cost effectiveness ratios (Additional file 1: ICER) based on fixed coverage bands (see above for the different interventions). Starting from zero coverage of all interventions, the first intervention to be implemented at the lowest coverage band was selected as that with the lowest ICER. At each subsequent step, either one of the existing interventions was increased to the next coverage band, or an additional intervention was introduced at the lowest coverage band, with the choice based on the option with the lowest ICER. This process was continued for each nominal budget until either all interventions were at maximum coverage or elimination was achieved.

Results at each budget are presented as the mean coverage of each intervention across 20 uncertainty runs that include model parameter (intervention impact) and cost uncertainty. As interventions coverage was based on a small number of fixed discrete bands, uncertainty intervals for this metric could not be calculated.

## Results

**Fig. 1:**
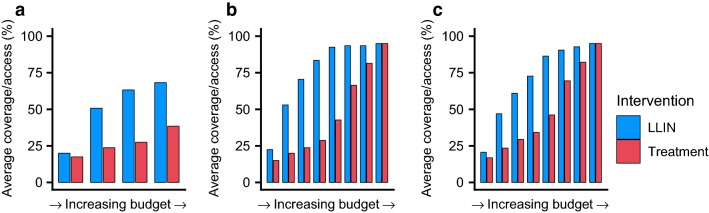
Cost-effective prioritization of LLINs and treatment. The average cost-effective scale-up of access to LLINs (blue bars) and coverage of treatment (red bars) for **a** low (baseline PfPr_2-10_: 10%), **b** medium (baseline PfPr_2-10_: 30%) and **c** high (baseline PfPr_2-10_: 60%) non-seasonal transmission settings. Outcomes are similar for the seasonal setting (Additional file 2: Figure S1). Coverage does not reach 100% in the low-transmission scenario as elimination is achieved

Figure 1 shows the most cost-effective pathways for scaling up interventions for three different initial transmission settings: low (panel A), medium (panel B) and high (panel C) initial parasite prevalence, respectively. As the budget is increased (shown on the x-axis), the mean coverage of two core interventions (LLINs and treatment) across all uncertainty runs is shown in the blue and red bars, respectively.

**Fig. 2:**
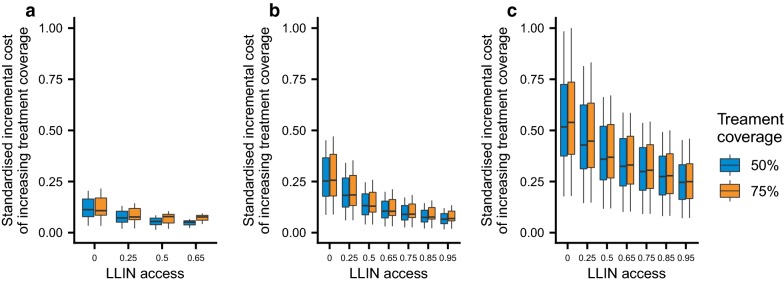
The standardized marginal cost of increasing treatment coverage. Treatment coverage is increased from 0 to 50% (blue boxes) or 75% (orange boxes) with respect to LLIN access. Increasing LLIN coverage prevents cases and therefore reduces the cost of increasing treatment coverage. In **a** low (baseline PfPr_2-10_: 10%) and **b** medium (baseline PfPr_2-10_: 30%) transmission settings the marginal impact of scaling LLINs decreases and treatment becomes a relatively cost-effective choice before coverage of LLINs is maximized. In the **c** high (baseline PfPr_2-10_: 60%) transmission settings transmission continues to decrease even as LLINs reach very high levels of coverage

Across all settings, LLINs are estimated to be the most cost-effective intervention and are therefore scaled first in the majority of simulation runs (Fig. 1, Additional file 2: Figure S1). In both medium and high transmission settings, achieving high levels of LLIN coverage prior to scaling-up treatment is estimated to be the most efficient. This is because LLIN coverage works synergistically with treatment coverage scale-up by reducing disease burden and therefore the number of treatments that are required (Fig. 2). This effect is most pronounced at high transmission levels where increasing LLIN coverage always leads to substantial reductions in transmission (and therefore cases that need to be treated). In the low transmission setting, treatment is introduced at a lower level of LLIN coverage. In this setting the marginal benefit of increasing LLIN coverage, quantified in terms of reduced treatment costs due to fewer cases, diminished with increasing LLIN coverage (Fig. 2a), at which point introducing or scaling treatment coverage becomes relatively more cost effective. Examples of posterior draws for the non-linear costs are shown in Additional file 9: Figure S8.

**Fig. 3:**
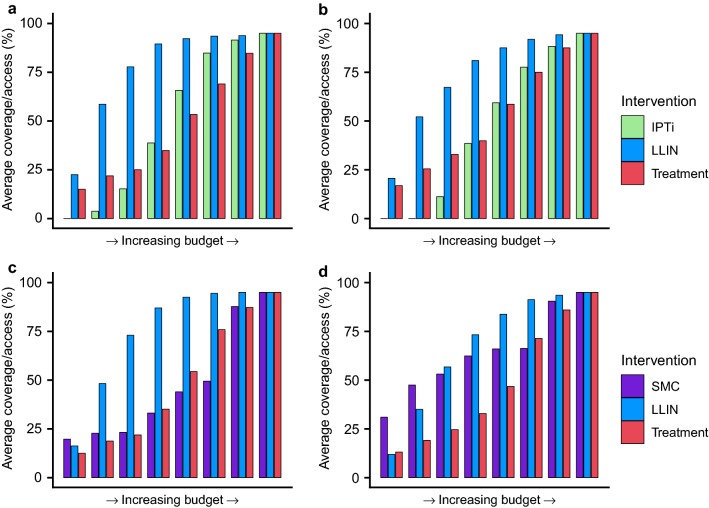
Cost-effective prioritization of LLINs, treatment and IPTi or SMC. The average cost-effective scale-up of access to LLINs (blue bars) and coverage of treatment (red bars) with IPTi (light green bars) in perennial transmission settings or SMC (purple bars) in seasonal transmission settings. Scale-up is shown for **a**, **c** medium (baseline PfPr_2-10_: 30%) and **b**, **d** high (baseline PfPr_2-10_: 60%) transmission settings

Figure 3 shows the same prioritization curves with the addition of SMC (in seasonal settings) or IPTi (in non-seasonal settings). In both medium and high transmission settings in which these interventions are recommended, chemoprevention is scaled up after scale-up of LLINs to high coverage levels. Introduction (especially of SMC) is selected earlier in the high transmission settings (Fig. 3b, d), driven by the concentration of cases in younger age groups at higher transmission levels. In the medium transmission setting (Fig. 3a, c) treatment is selected alongside SMC as cases are less concentrated in the younger age group.

**Fig. 4:**
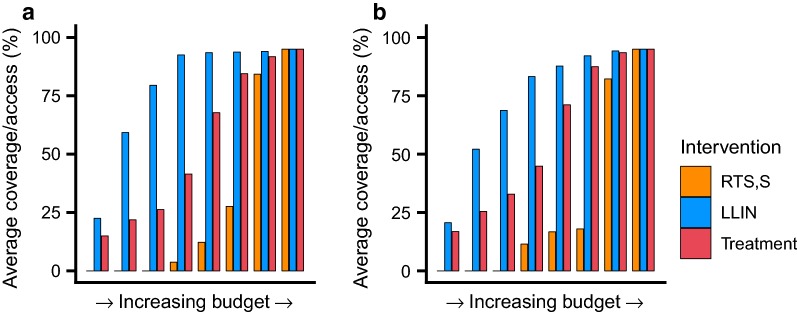
Cost-effective prioritization of LLINs, treatment and the RTS,S vaccine. The average cost-effective scale-up of access to LLINs (blue bars), coverage of treatment (red bars) and the RTS,S vaccine (orange bars) for **a** medium (baseline PfPr_2-10_: 30%) and **b** high (baseline PfPr_2-10_: 60%) perennial transmission settings

Figure 4 shows how the introduction of the RTS,S vaccine (at an assumed cost of $5 per dose) compares as an alternative disease prevention method to SMC or IPTi. In all settings the vaccine is selected late in the cost-effective pathway (Fig. 4). Whilst the vaccine remains a cost-effective measure [13], it is relatively less cost effective than LLINs or treatment and hence enters the prioritization pathway after these two interventions.

**Fig. 5:**
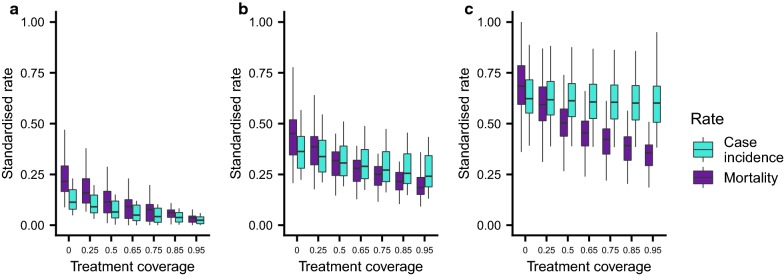
The relative impact of treatment coverage on case incidence and mortality rates. In the **a** low (baseline PfPr_2-10_: 10%) transmission setting increasing treatment coverage leads to reductions in the mortality rate and incidence. In the **b** medium (baseline PfPr_2-10_: 30%) and **c** high (baseline PfPr_2-10_: 60%) transmission settings increasing treatment coverage is still associated with declines in the mortality rate. However, as transmission increases the impact of increasing treatment coverage on incidence becomes less. For all examples LLIN access is fixed at 25%

Scale-up trends are broadly similar for the alternative outcome measure of clinical incidence only (Additional file 3: Figure S2, Additional file 4: Figure S3, Additional file 5: Figure S4) or a weighted combination of incidence and mortality rates only (Additional file 6: Figure S5, Additional file 7: Figure S6, Additional file 8: Figure S7, Additional file 9: Fig. S8). When maximizing deaths averted, the drug-based strategies (treatment and chemoprevention) are introduced earlier in the pathway as they impact the rate of progression from clinical disease to severe disease and death. Increasing treatment coverage has a positive impact on mortality rate, regardless of the transmission setting (Fig. 5). Treatment has a relatively larger impact on case incidence in the lower transmission setting, where infections are more likely to be symptomatic and lead to treatment seeking. In high transmission settings, the small impact on transmission that clearing cases (and the subsequent prophylactic period) has is outweighed by the high force of infection.

## Discussion

A mathematical model of malaria transmission, combined with costing data was used to estimate the most cost-effective prioritization of combination of malaria interventions including treatment, LLINs, SMC, iPTI, and the RTS,S vaccine.

At the lowest budget levels, the modelling indicates that implementation and scaling prevention, in the form of vector control (LLINs) is the most cost-effective option. With increasing transmission, scaling prevention also has an important role of reducing the burden of malaria upon the routine health system. As budgets increase to moderate levels, treatment coverage is scaled up. As the nominal budget is increased further, both LLINs and treatment are scaled up together. The cost of both LLINs and treatment is modelled to rise non-linearly at higher coverage. This reflects inefficiencies and challenges associated with attaining very high access and coverage of LLINs and treatment. As the budget is increased, the point at which an intervention is scaled is therefore sensitive to the relative inflection points in the cost curves. The switch from LLINs to treatment is, in part, driven by the increasing marginal costs of pushing LLIN access to mid to high levels. Likewise, attaining the maximum coverage of treatment is assumed to be increasingly expensive (as health system capacity, scope and scale must be increased) and therefore the final steps where LLIN access and treatment coverage are both very high, are increasingly expensive. The impact and cost-effectiveness of scaling treatment, as well as being influenced by preventative interventions, is also a complex function of the immune dynamics of a population which impact the likelihood an infection is symptomatic and would instigate treatment-seeking. In this instance, it was assumed that all populations are equally likely to access either intervention. Spatial heterogeneities in the distributions of both malaria and available interventions could negatively impact outcomes (by decreasing impact, increasing costs or both). By assessing the impact of intervention packages at equilibrium in the model, temporal factors were omitted that may differ between interventions. These could include the time-scale for scale-up and associated impact. As such, policy decisions would also need to consider the feasibility and logistics of scaling any specific intervention to a target coverage. It is likely that the ability to execute an intervention package in a timely manner will also vary with the target coverage and where there is little to distinguish between two or more packages of interventions this may be a critical factor in decision making. As such the impact of timing and dynamic shifts in transmission is an important open research question in this field.

Whilst prevention proved more cost-effective using this approach, there are ethical considerations surrounding providing treatment for all that fit with the broader picture of universal health coverage more generally [30]. The goal of strengthening the health system, and therefore being better able to achieve targets for universal health coverage (UHC) is likely to work in synergy with reducing the health system burden through prevention of malaria in affected countries.

IPTi and SMC are further cost-effective tools at the disposal of malaria control programmes in regions where their use is appropriate. Both chemoprevention options were introduced and scaled up after LLINs had reached mid to high levels of coverage, being best layered in addition to core vector control as opposed to replacing it. The setting was influential here also, as with increased intensity of transmission, cases become more concentrated within younger age groups [31]. Therefore, the relative cost-effectiveness of these child- and infant-focussed strategies (particularly SMC, but also IPTi) becomes greater at higher levels of transmission.

The potential for the RTS,S vaccine to be included as an additional new tool in medium and high transmission areas was also examined. Whilst still cost-effective, and in line with previous work [32], the vaccine was introduced later than for other interventions. This is driven by the limited age groups the vaccine provides protection to combined with the relatively high costs (at an assumed cost per dose of $5). It is, therefore, apparent that focussing resources on achieving good levels of vector control and treatment coverage would be the priority before investing in vaccination.

Surveillance, monitoring and evaluation activities were not included in this analysis despite the importance of these components to a malaria control programme. Other interventions, for example, behaviour change communication [33], could also modify the cost-curve gradient. Data on the cost of these components at varying levels of ‘coverage’ is scarce and their impact is highly dependent on the mix of interventions and level of heterogeneity in transmission. There are, therefore, a range of benefits, economic and otherwise, that a well-functioning surveillance, monitoring and evaluation programme can bring about that are not captured here.

The outputs presented here demonstrate averaged approaches for generic transmission settings, aimed at informing decision-making in the general case. In practice there will be layers of context-specific information that will influence and shape the decision-making process. Malaria control programmes are faced with optimizing the impact of a single budget across an area with heterogeneous transmission, leading to a more complex optimization problem than presented here, and other more sophisticated approaches including, for example, simulated annealing [34] have been proposed for these scenarios. Some costs, for example, inpatient and outpatient costs, vary a great deal between countries, impacting country-specific treatment coverage. As discussed previously, the local ecology, vector species and associated bionomics will impact the choice of vector control. Insecticide resistance, where known, will strongly influence vector control decisions. This decision is further influenced by other factors: the logistics associated with large-scale programmatic deployment of IRS, historical precedents and acceptance. Drug resistance plays a key role in determining the application of chemoprevention strategies. Whilst the uncertainty propagated through this analysis reflects this between county variation, the interpretation must still be made on a country-by-country basis. Key drivers of much uncertainty are the non-commodity costs (e.g., delivery costs), which are likely to vary between countries substantially. Better knowledge of these costs would improve cost-effective decision-making.

The ICER was used as the decision-step making tool in this analysis. It should be stated that this step-wise approach could potentially result in a non-optimal solution for any given budget. This is a result of taking a step-wise approach in a highly non-linear environment (both with respect to impact and cost) leading to restriction of the solution to local optima. The ICER does however benefit from simple interpretation and implementation.

## Conclusions

This study reiterates the importance of a combination of prevention, via vector control (here LLINs), and treatment as the cornerstone of malaria control in SSA. Additional currently recommended interventions (chemoprevention) and tools in development (RTS,S) will be valuable in those many areas where vector control and treatment are themselves not enough to bring about a transition to elimination. When resources are limited the prioritization of the use of available tools can help to maximize the impact of available finances, reducing malaria morbidity and mortality in a cost-effective manner.

## Additional files

**Additional file 1.** Additional information.

**Additional file 2: Figure S1.** Cost-effective prioritisation of LLINs and treatment. The average cost-effective scale-up of access to LLINs (blue bars) and coverage of treatment (red bars) for A) low (baseline PfPr_2-10_: 10%), B) medium (baseline PfPr_2-10_: 30%) and C) high (baseline PfPr_2-10_: 60%) seasonal transmission settings.

**Additional file 3: Figure S2.** Cost-effective prioritisation of LLINs and treatment: Cases only outcome. The average cost-effective scale-up of access to LLINs (blue bars) and coverage of treatment (red bars) for A) low (baseline PfPr_2-10_: 10%), B) medium (baseline PfPr_2-10_: 30%) and C) high (baseline PfPr_2-10_: 60%) perennial transmission settings.

**Additional file 4: Figure S3.** Cost-effective prioritisation of LLINs, treatment and IPTi or SMC: Cases only outcome. The average cost-effective scale-up of access to LLINs (blue bars) and coverage of treatment (red bars) with IPTi (light green bars) in perennial transmission settings or SMC (purple bars) in seasonal transmission settings. Scale-up is shown for A, C) medium (baseline PfPr_2-10_: 30%) and B, D) high (baseline PfPr_2-10_: 60%) transmission settings.

**Additional file 5: Figure S4.** Cost-effective prioritisation of LLINs, treatment and the RTS,S vaccine: Cases only outcome. The average cost-effective scale-up of access to LLINs (blue bars), coverage of treatment (red bars) and the RTS,S vaccine (orange bars) for A) medium (baseline PfPr_2-10_: 30%) and B) high (baseline PfPr_2-10_: 60%) perennial transmission settings.

**Additional file 6: Figure S5.** Cost-effective prioritisation of LLINs and treatment: Cases and deaths outcome. The average cost-effective scale-up of access to LLINs (blue bars) and coverage of treatment (red bars) for A) low (baseline PfPr_2-10_: 10%), B) medium (baseline PfPr_2-10_: 30%) and C) high (baseline PfPr_2-10_: 60%) perennial transmission settings.

**Additional file 7: Figure S6.** Cost-effective prioritisation of LLINs, treatment and IPTi or SMC: Cases and deaths outcome. The average cost-effective scale-up of access to LLINs (blue bars) and coverage of treatment (red bars) with IPTi (light green bars) in perennial transmission settings or SMC (purple bars) in seasonal transmission settings. Scale-up is shown for A, C) medium (baseline PfPr_2-10_: 30%) and B, D) high (baseline PfPr_2-10_: 60%) transmission settings.

**Additional file 8: Figure S7.** Cost-effective prioritisation of LLINs, treatment and the RTS,S vaccine: Cases and deaths outcome. The average cost-effective scale-up of access to LLINs (blue bars), coverage of treatment (red bars) and the RTS,S vaccine (orange bars) for A) medium (baseline PfPr_2-10_: 30%) and B) high (baseline PfPr_2-10_: 60%) perennial transmission settings.

**Additional file 9: Figure S8.** Example of posterior draws for the non-linear component of treatment delivery costs. The cost multiplier is applied to the cost of treating a clinical case to provide an estimate of distribution costs that increase non-linearly as coverage reaches very high levels. Assumes that distribution at baseline (in the absence of non-linear effects) is approximately 15% of the cost per clinical case.

## Abbreviations

ACT: artemisinin-based combination therapy
EPI: expanded programme of immunization
GTS: Global Technical Strategy for malaria
ICER: incremental cost effectiveness ratio
IPTi: intermittent preventive treatment in infants
IPTp: intermittent preventive treatment for pregnant women
IRS: indoor residual spraying
ITN: insecticide-treated bed net
LLINs: long-lasting insecticide-treated bed nets
PMI: President’s Malaria Initiative
RDT: rapid diagnostic test
SMC: seasonal malaria chemoprevention
SP: sulfadoxine-pyrimethamine
SP-AQ: sulfadoxine-pyrimethamine + amodiaquine
SSA: sub-Saharan Africa
UHC: universal health coverage
WHO: World Health Organization
