# Supplementary material for: Prioritizing the scale-up of interventions for malaria control and elimination
Source: Malar J. 2019 Apr 8;18:122. doi: 10.1186/s12936-019-2755-5 (PMC6454681; doi:10.1186/s12936-019-2755-5)
Supplement: Supplementary file 1 — Additional file 1. Additional information. [file 12936_2019_2755_MOESM1_ESM.docx]

# Additional information

**Figure S1 Cost-effective prioritisation of LLINs and treatment.** The average cost-effective scale-up of access to LLINs (blue bars) and coverage of treatment (red bars) for A) low (baseline PfPr_2-10_: 10%), B) medium (baseline PfPr_2-10_: 30%) and C) high (baseline PfPr_2-10_: 60%) seasonal transmission settings.

## Alternative outcome measures

### Clinical incidence

**Figure S2 Cost-effective prioritisation of LLINs and treatment: Cases only outcome.** The average cost-effective scale-up of access to LLINs (blue bars) and coverage of treatment (red bars) for A) low (baseline PfPr_2-10_: 10%), B) medium (baseline PfPr_2-10_: 30%) and C) high (baseline PfPr_2-10_: 60%) perennial transmission settings.

**Figure S3 Cost-effective prioritisation of LLINs, treatment and IPTi or SMC: Cases only outcome.** The average cost-effective scale-up of access to LLINs (blue bars) and coverage of treatment (red bars) with IPTi (light green bars) in perennial transmission settings or SMC (purple bars) in seasonal transmission settings. Scale-up is shown for A, C) medium (baseline PfPr_2-10_: 30%) and B, D) high (baseline PfPr_2-10_: 60%) transmission settings.

**Figure S4 Cost-effective prioritisation of LLINs, treatment and the RTS,S vaccine: Cases only outcome.** The average cost-effective scale-up of access to LLINs (blue bars), coverage of treatment (red bars) and the RTS,S vaccine (orange bars) for A) medium (baseline PfPr_2-10_: 30%) and B) high (baseline PfPr_2-10_: 60%) perennial transmission settings.

### Equally weighted sum of clinical incidence and mortality rate

**Figure S5 Cost-effective prioritisation of LLINs and treatment: Cases and deaths outcome.** The average cost-effective scale-up of access to LLINs (blue bars) and coverage of treatment (red bars) for A) low (baseline PfPr_2-10_: 10%), B) medium (baseline PfPr_2-10_: 30%) and C) high (baseline PfPr_2-10_: 60%) perennial transmission settings.

**Figure S6 Cost-effective prioritisation of LLINs, treatment and IPTi or SMC: Cases and deaths outcome.** The average cost-effective scale-up of access to LLINs (blue bars) and coverage of treatment (red bars) with IPTi (light green bars) in perennial transmission settings or SMC (purple bars) in seasonal transmission settings. Scale-up is shown for A, C) medium (baseline PfPr_2-10_: 30%) and B, D) high (baseline PfPr_2-10_: 60%) transmission settings.

**Figure S7 Cost-effective prioritisation of LLINs, treatment and the RTS,S vaccine: Cases and deaths outcome.** The average cost-effective scale-up of access to LLINs (blue bars), coverage of treatment (red bars) and the RTS,S vaccine (orange bars) for A) medium (baseline PfPr_2-10_: 30%) and B) high (baseline PfPr_2-10_: 60%) perennial transmission settings.

## ICER

The incremental cost effectiveness ratio is defined as

$\text{ICER} =\frac{C_{1}-C_{0}}{E_{1}-E_{0}}$ (1.1)

where C_1_ and E_1_ are the cost and effect of the next step and C_0_ and E_0_ the cost and effect of the current step. The next step chosen is the one which minimised the ICER.

## Non-linear fits

The non-linear components of treatment and LLIN costs are represented with the following hill function

$y=b\left(\frac{1}{1-\frac{\mathrm{c}}{c_{\max}}}-1\right)^{\frac{1}{a}}$ , (1.2)

where c is coverage c_max_ the maximum attainable coverage (assumed to be 1), and a and b parameters determining the shape and slope of the curve. Parameters a and b are fitted to the modelled estimate of required nets per capita [14] in the case of LLINs, simulated linear data (up to 46% coverage) in the case of treatment and surveillance cost per person (assuming coverage of 46%) using Metropolis–Hastings MCMC methods. We use uninformative uniform priors for a and b for all fits. The resultant uncertainty being taken from 20 draws from the posterior distribution.

**Figure S8. Example of posterior draws for the non-linear component of treatment delivery costs.** The cost multiplier is applied to the cost of treating a clinical case to provide an estimate of distribution costs that increase non-linearly as coverage reaches very high levels. Assumes that distribution at baseline (in the absence of non-linear effects) is approximately 15% of the cost per clinical case.

## Public private sector treatment

Public/Private sector treatment splits were based on the most recent DHS survey data [30] and based on a recoding of responses to the question “Place first sought treatment for fever”.
