# Supplementary figures and images for: Prioritizing the scale-up of interventions for malaria control and elimination
Source: Malar J. 2019 Apr 8;18:122. doi: 10.1186/s12936-019-2755-5 (PMC6454681; doi:10.1186/s12936-019-2755-5)

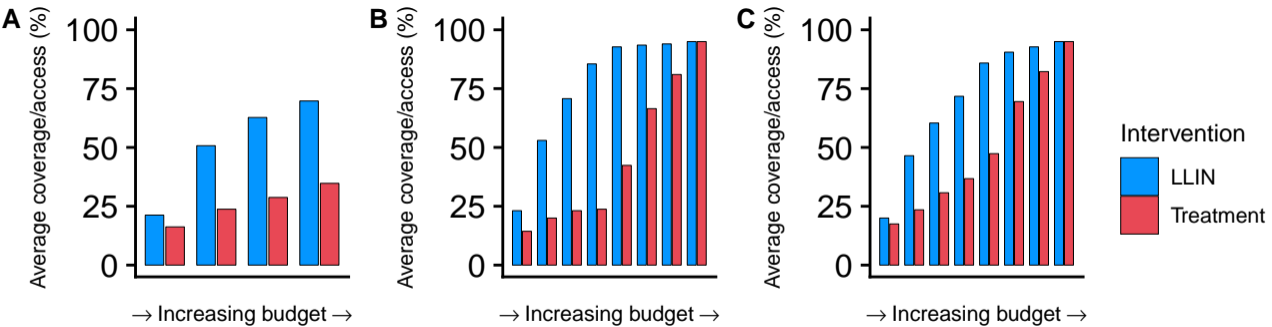

Supplement: Supplementary file 2 — Additional file 2: Figure S1. Cost-effective prioritisation of LLINs and treatment. The average cost-effective scale-up of access to LLINs (blue bars) and coverage of treatment (red bars) for A) low (baseline PfPr2-10: 10%), B) medium (baseline PfPr2-10: 30%) and C) high (baseline PfPr2-10: 60%) seasonal transmission settings. [file 12936_2019_2755_MOESM2_ESM.pdf]

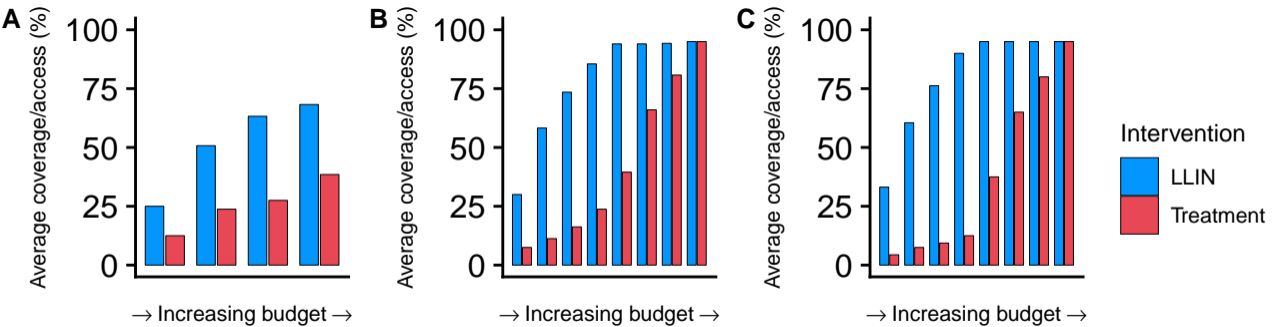

Supplement: Supplementary file 3 — Additional file 3: Figure S2. Cost-effective prioritisation of LLINs and treatment: Cases only outcome. The average cost-effective scale-up of access to LLINs (blue bars) and coverage of treatment (red bars) for A) low (baseline PfPr2-10: 10%), B) medium (baseline PfPr2-10: 30%) and C) high (baseline PfPr2-10: 60%) perennial transmission settings. [file 12936_2019_2755_MOESM3_ESM.pdf]

**A**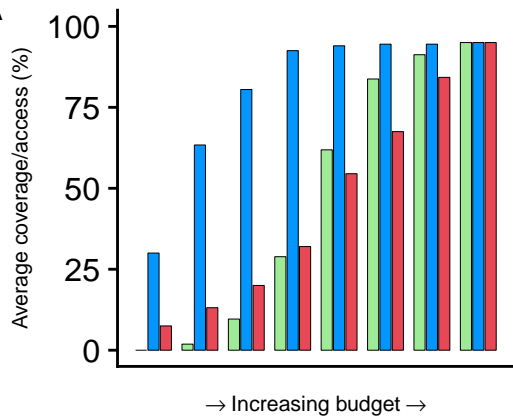**B**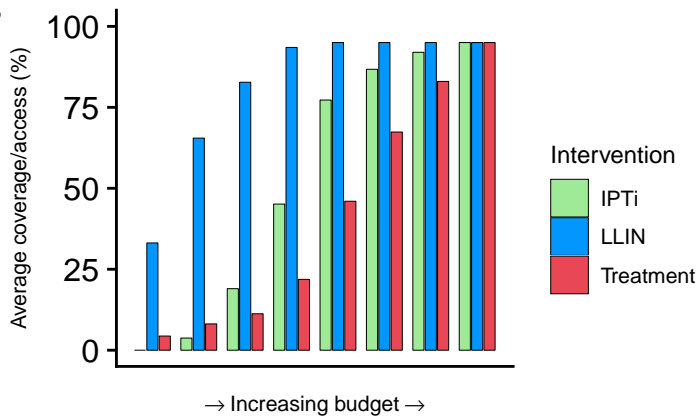**C**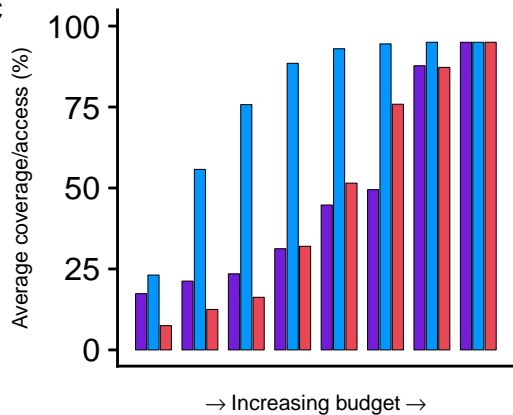**D**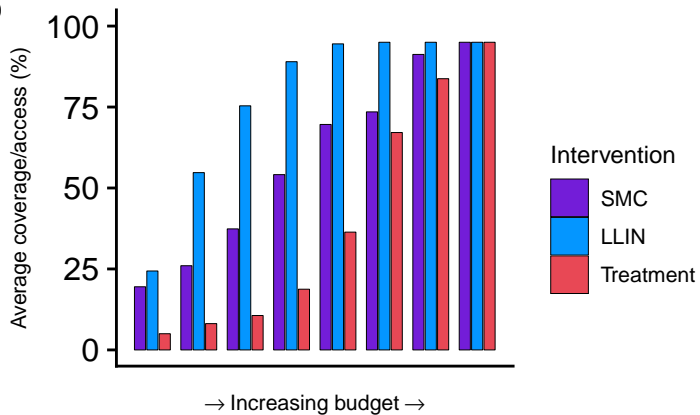

Supplement: Supplementary file 4 — Additional file 4: Figure S3. Cost-effective prioritisation of LLINs, treatment and IPTi or SMC: Cases only outcome. The average cost-effective scale-up of access to LLINs (blue bars) and coverage of treatment (red bars) with IPTi (light green bars) in perennial transmission settings or SMC (purple bars) in seasonal transmission settings. Scale-up is shown for A, C) medium (baseline PfPr2-10: 30%) and B, D) high (baseline PfPr2-10: 60%) transmission settings. [file 12936_2019_2755_MOESM4_ESM.pdf]

**A**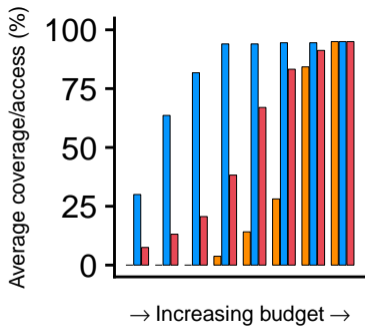**B**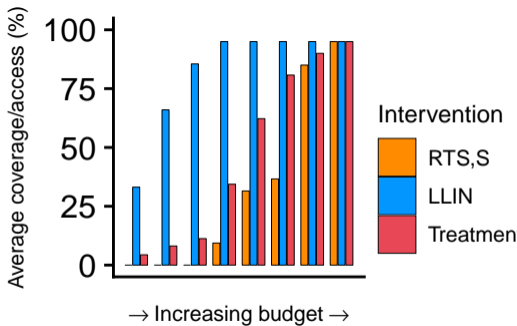

Supplement: Supplementary file 5 — Additional file 5: Figure S4. Cost-effective prioritisation of LLINs, treatment and the RTS,S vaccine: Cases only outcome. The average cost-effective scale-up of access to LLINs (blue bars), coverage of treatment (red bars) and the RTS,S vaccine (orange bars) for A) medium (baseline PfPr2-10: 30%) and B) high (baseline PfPr2-10: 60%) perennial transmission settings. [file 12936_2019_2755_MOESM5_ESM.pdf]

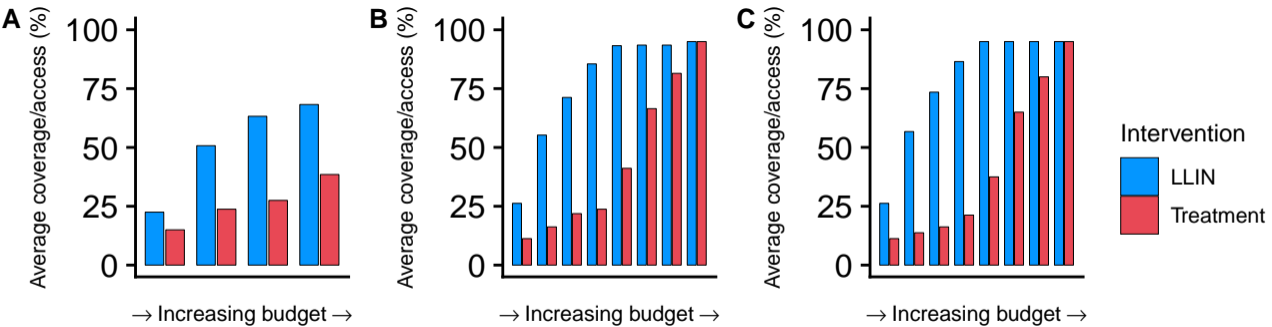

Supplement: Supplementary file 6 — Additional file 6: Figure S5. Cost-effective prioritisation of LLINs and treatment: Cases and deaths outcome. The average cost-effective scale-up of access to LLINs (blue bars) and coverage of treatment (red bars) for A) low (baseline PfPr2-10: 10%), B) medium (baseline PfPr2-10: 30%) and C) high (baseline PfPr2-10: 60%) perennial transmission settings. [file 12936_2019_2755_MOESM6_ESM.pdf]

**A**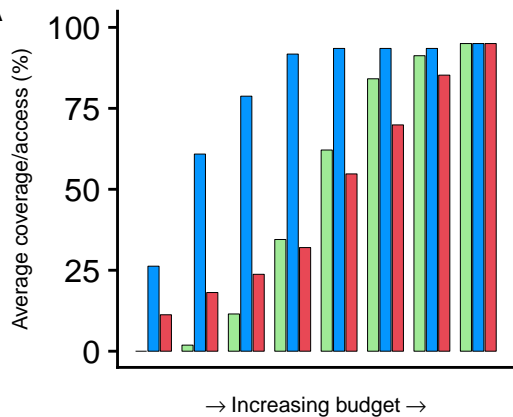**B**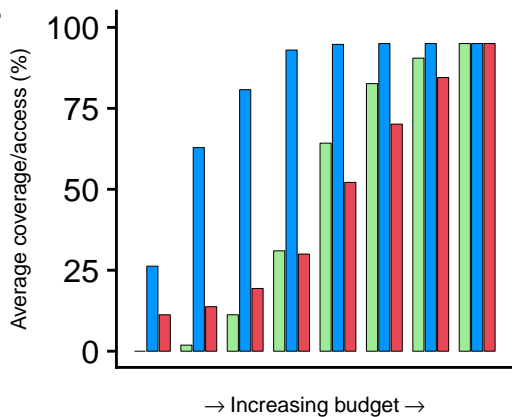

Intervention

IPTi

LLIN

Treatment

**C**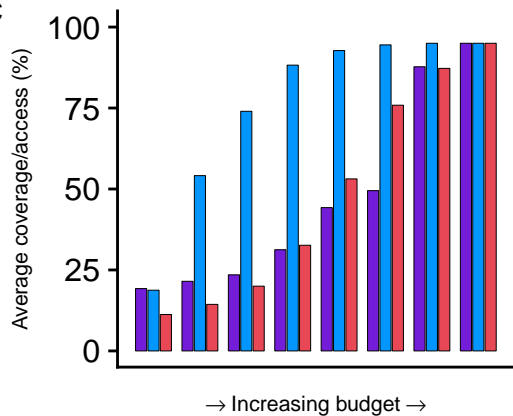**D**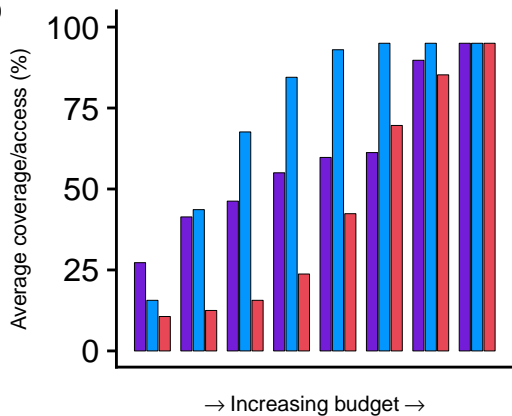

Intervention

SMC

LLIN

Treatment

Supplement: Supplementary file 7 — Additional file 7: Figure S6. Cost-effective prioritisation of LLINs, treatment and IPTi or SMC: Cases and deaths outcome. The average cost-effective scale-up of access to LLINs (blue bars) and coverage of treatment (red bars) with IPTi (light green bars) in perennial transmission settings or SMC (purple bars) in seasonal transmission settings. Scale-up is shown for A, C) medium (baseline PfPr2-10: 30%) and B, D) high (baseline PfPr2-10: 60%) transmission settings. [file 12936_2019_2755_MOESM7_ESM.pdf]

**A**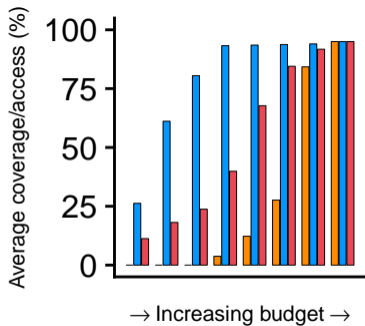**B**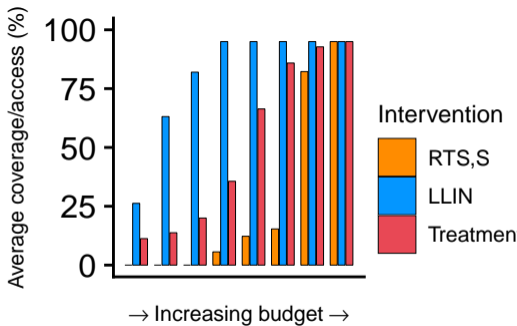

Supplement: Supplementary file 8 — Additional file 8: Figure S7. Cost-effective prioritisation of LLINs, treatment and the RTS,S vaccine: Cases and deaths outcome. The average cost-effective scale-up of access to LLINs (blue bars), coverage of treatment (red bars) and the RTS,S vaccine (orange bars) for A) medium (baseline PfPr2-10: 30%) and B) high (baseline PfPr2-10: 60%) perennial transmission settings. [file 12936_2019_2755_MOESM8_ESM.pdf]

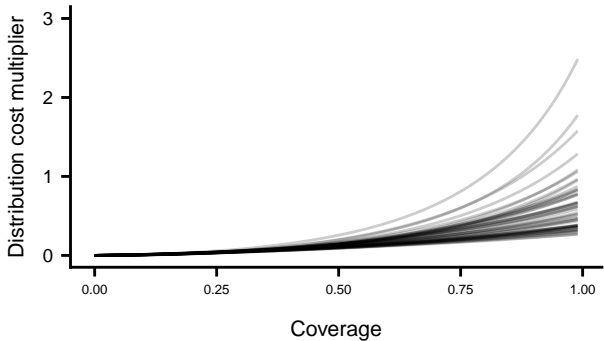

Supplement: Supplementary file 9 — Additional file 9: Figure S8. Example of posterior draws for the non-linear component of treatment delivery costs. The cost multiplier is applied to the cost of treating a clinical case to provide an estimate of distribution costs that increase non-linearly as coverage reaches very high levels. Assumes that distribution at baseline (in the absence of non-linear effects) is approximately 15% of the cost per clinical case. [file 12936_2019_2755_MOESM9_ESM.pdf]
